# Effects of Vibration in Forced Posture on Biochemical Bone Metabolism Indices, and Morphometric and Mechanical Properties of the Lumbar Vertebra

**DOI:** 10.1371/journal.pone.0078640

**Published:** 2013-11-12

**Authors:** Qi Chang, Fuling Wei, Li Zhang, Xiaowei Ju, Lvgang Zhu, Changlin Huang, Tao Huang, Xincheng Zuo, Chunfang Gao

**Affiliations:** 1 Institute of Military Training Related Medical Science, The 150th Hospital of PLA, Luoyang, Henan, China; 2 Department of Cardiosurgery, No. 309 Hospital of PLA, Beijing, China; 3 Institute of Anus and Intesine, The 150th Hospital of PLA, Luoyang, Henan, China; Faculté de médecine de Nantes, France

## Abstract

Epidemiological studies have shown a relatively strong association between occupational lower back pain (LBP) and long-term exposure to vibration. However, there is limited knowledge of the impact of vibration and sedentariness on bone metabolism of the lumbar vertebra and the mechanism of bone-derived LBP. The aim of this study was to investigate the effects of vibration in forced posture (a seated posture) on biochemical bone metabolism indices, and morphometric and mechanical properties of the lumbar vertebra, and provide a scientific theoretical basis for the mechanism of bone-derived LBP, serum levels of Ca^2+^, (HPO4)^2−^, tartrate-resistant acid phosphatase (TRAP), bone-specific alkaline phosphatase (BALP), and bone gla protein (BGP),the pathological changes and biomechanics of lumbar vertebra of New Zealand white rabbits were studied. The results demonstrate that both forced posture and vibration can cause pathological changes to the lumbar vertebra, which can result in bone-derived LBP, and vibration combined with a seated posture could cause further damage to bone metabolism. Serological changes can be used as early markers for clinical diagnosis of bone-derived LBP.

## Introduction

Back pain is the second most common chronic condition, with a reported lifetime incidence of 84% in industrialized countries [1]. More than a third of patients visit orthopedic clinics due to lower back pain (LBP) [2]. In Western developed countries, LBP is a very important cause of disability and industrial injury indemnification, and 70%–80% of the population has suffered from LBP, with a prevalence of 30%. The relapse rate is very high and reaches 60–85% for patients with single LBP history, which causes great suffering in daily life [3].

Epidemiological studies have shown a relatively strong association between occupational LBP and long-term exposure to vibration [2], [4], [5], [6], [7], with the risk of injury increasing as the duration and dose of vibration increases [8]. For professional reasons, heavy equipment vehicle (HEV) operators, who regularly operate and maintain heavy construction equipment, including bulldozers, front-end loaders, rollers, backhoes, and so on, are continuously exposed to vibration and kept in a seated posture. Waters et al. found that HEV operators had more than twice the risk of developing LBP compared with non-HEV operators through a meta-analysis [9]. Yi et al. found that the leading causes of LBP were sedentariness (38.4%) and vibration (18.1%) in urban patients [2]. Okumnribido at al. have shown that interaction effects of exposure to two or more of vibration, posture (sitting), and manual material handling (MMH), rather than the effects of an individual exposure, are the main contributors to the precipitation of LBP [10]. Magora has suggested that occupational factors contributing to the acceleration of spinal degeneration include heavy physical loads, MMH, prolonged sitting, sustained non-neutral work postures, and vehicle driving [11]. Videman confirmed that occupational exposures can be viewed as the primary source of mechanical factors that damage the spine [12]. This finding was supported by Frymoyer, who found that gender, age, occupation, and exposure to vehicular vibration were all associated with the occurrence of LBP [13]. Thus increasing epidemiological studies have demonstrated that vibration is closely associated with LBP.

Bone tissue is the main store in the body of macro- and microelements, among which the most important are calcium (Ca), phosphorus (P), and magnesium (Mg), which provide high mineral density and mechanical endurance of the skeleton [14], [15]. Bone is a dynamic tissue possessing the ability to remodel throughout life. Bone formation and resorption coupled under normal circumstances are important processes, which dependent on the activity of osteoclasts and osteoblasts [16]. Optimal balance between bone formation and resorption is crucial to maintain the biochemical competence of the skeleton, its structural organization, strength, and function [17]. In postmenopausal osteoporosis, increased osteoclastic activity may be accompanied by increased, decreased, or unchanged osteoblastic activity [18]. Efforts have been made to detect circulating markers of bone cell activity. Bone-specific alkaline phosphatase (BALP) is considered the single most accurate marker of bone formation which has been suggested as the classic marker for increased osteoblastic activity in Paget's disease of bone [19]. Tartrate-resistant acid phosphatase (TRAP) has been used as an osteoclast marker and proposed as a serum marker for bone resorptive activity in pathological states such as osteoporosis [20]. Evaluation of biochemical bone turnover markers in plasma or serum provides useful information on bone metabolic processes within the skeleton [21]. However, there is limited knowledge of the impact of vibration and sedentariness on bone metabolism and the mechanism of bone-derived LBP. The aim of this study was to investigate the effect of vibration in forced posture (a seated posture) on the metabolism of the lumbar vertebra, and provide a scientific theoretical basis for the mechanism of bone-derived LBP.

## Materials and Methods

### Ethics Statement

he experimental procedures used in this study were approved by the Local Ethics Committee on Animal Experimentation of the Military Training Medicine Institute in the 150^th^ Hospital of PLA in Luoyang, Henan, China.

### Animals and grouping

**Figure 1:**
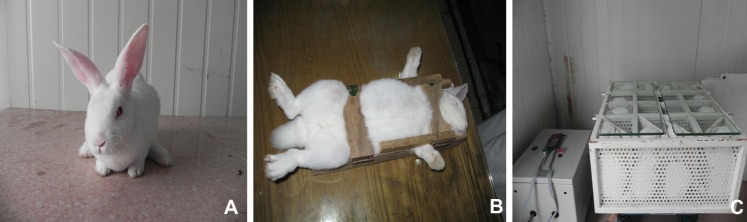
Construction of model of vibration in a seated posture. (A) The New Zealand white rabbit in a normal state. (B) Construction of a seated posture. (C) The animal model of vibration in a seated posture.

**Figure 2:**
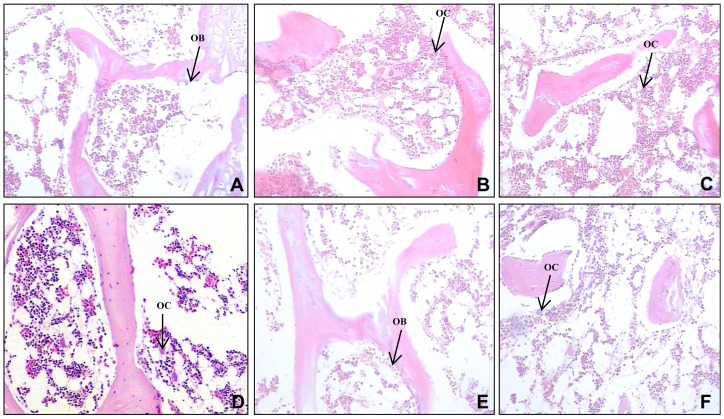
Histological assessment of the fifth lumbar vertebra (L5) (HE, 10×40). (A) Osteoblasts arranged in a monolayer around the bone trabecula, osteoclasts are rare (control at 6 weeks). (B) Osteoblasts are decreased, osteoclast are increased, bone trabecula and bone structure are damaged (VG at 2 weeks). (C) Osteoclasts are further increased, osteoblasts continued to be significantly reduced, increased empty bone lacuna rate, decreased bone trabecula, and structural disorder (VG at 4 weeks). (D) Osteoblasts are markedly decreased, osteoclasts are visible, marrow cavity is filled with fat cells (VG at 4 weeks). (E) Bone structure is improved, osteoblasts are slightly increased, and osteoclasts are rare (VG at 6 weeks compared with VG at 2 and 4 weeks). (F) Osteoblasts are decreased, osteoclasts are increased, and structure of the bone trabecula is damaged (PG at 6 weeks). OB denotes osteoblasts and OC denotes osteoclast.

One hundred and twenty-six New Zealand white rabbits (both male and female, body weight 2.5–3.0 kg, mean 2.8 kg, 10–12 months of age) provided by the Military Training Medicine Institute in the 150^th^ Hospital of PLA, Luoyang, Henan, China were randomly divided into three groups: untreated control group (Control, *n* = 42), animals allowed unrestricted activity in the cage, untreated by any intervention processing. Forced posture group (PG, *n* = 42), the trunk was fastened by a board in the back and belted by rope, and the upper limbs were also fastened by short stick (Fig. 1B). To mimic the driver's posture, animals kept in a seated posture in a special cage for 2 hours per day, 5 days a week. Vibration group (VG, *n* = 42,), animals received the same treatment as above were kept in a seated posture and vibrated at 4 Hz (give the rabbit a 4 HZ syntony and 0.7 mm amplitude) by an electro-dynamic vibration machine (Hongda instrument co., LTD, Tianjin, China) for 2 hours per day, 5 days a week (Fig. 2C). The accuracy of the vibration was controlled by electronic control.

### Serum assessments

Blood was collected from the heart of all the rabbits in the groups at the 2^nd^, 4^th^, and 6^th^ weekend. 5 ml blood was drawn into the vacuum cleaner procoagulant tube, and serum was separated by centrifugation at 4°C, 2500 r/min for 15 min. 1 ml serum was used for measuring levels of Ca^2+^ and (HPO4)^2−^ using the Olympus chemistry analyzer (Olympus, USA) at the 2^nd^, 4^th^, and 6^th^ weekend. The remaining serum was stored at −70°C and used for measuring the activity of tartrate-resistant acid phosphatase (TRAP), bone-specific alkaline phosphatase (BALP), and bone gla protein (BGP). The activity of TRAP, BALP, and BGP was assessed using commercially available ELISA kits (interassay CV<10%; R&D Company, USA). All samples were assayed in duplicate.

### HE staining and cell counting

The fifth lumbar vertebra (L5) was decalcified in 10% EDTA in 0.01 M phosphate buffer (pH 7.4) at a temperature of 4°C for 1 week. It was then dehydrated in a graded series of ethanol solutions at 4°C, embedded in paraffin, and sectioned at a thickness of 5 μm. The sections were processed and stained with HE [22]. Cell number in a field of 500 μm×500 μm was counted. Five fields per section were randomly selected at ×400 magnification and photographed for counting the number of cells. The analyses were performed in a blinded manner by two pathologists, Q Chang and L Zhou.

### Biomechanical testing

For biomechanical testing, rabbits were randomly divided into five groups: untreated control group (Control, *n* = 18), animals allowed unrestricted activity in the cage, untreated by any intervention processing; forced posture group (PG, *n* = 18), animals kept in a seated posture in a special cage for 2 hours per day, 5 days a week; animals kept in a seated posture and vibrated at 4 Hz (4 Hz, *n* = 18), or 5 Hz (5 Hz, *n* = 18), or 6 Hz (5 Hz, *n* = 18), for 2 hours per day, 5 days a week. Six New Zealand white rabbits were randomly selected from each group and sacrificed at the 2^nd^, 4^th^, and 6^th^ weekend, and the fourth lumbar vertebra (L4) was obtained for biomechanical testing. After sacrifice, L4 was hermetically stored at −20°C until use. The stored bones were thawed at 4°C, immersed in saline, and used immediately for the biomechanical test. The trunk of the lumbar was separated and made into a column contour for the compression test. The biomechanical test was performed in a computerized material testing system (EZ Test; Shimadzu Co., Kyoto, Japan). The lumbar vertebrae were placed on the smooth surface of a steel disk and axially compressed by the smooth surface of a steel rod attached to a load cell at a constant speed of 2 mm/min. The force-deflection curve was displayed on a monitoring recorder linked to the tester for each specimen. Maximum load and structural stiffness were obtained from the compression tests of vertebral bodies.

### Statistical analysis

SPSS 16.0 (SPSS Inc, Chicago, IL). was used for statistical analysis. Data were expressed as the mean ± standard deviation (SD). The statistical significance of differences in the mean value between different groups was determined by Mann-Whitney U-test. Data were considered statistically significant when *P*<0.05.

## Results

### Change in serum Ca^2+^ and (HPO4)^2−^

Compared with the control group (3.19±0.15 mmol/l), levels of Ca^2+^ in the VG group were significantly increased at the fourth week (3.49±0.11 mmol/l, *P* = 0.0317), and levels in the PG group (3.32±0.18 mmol/l) were significantly higher than in control (3.24±0.30 mmol/l, *P* = 0.0253) at the sixth week. There was no difference in levels of serum (HPO4)^2−^ between the different groups at any time point (all *P* = 0.094) (Table 1).

**Table 1:** Change in serum Ca^2+^ and P^2+^ in the groups (mean *±* SD, *n* = 42).

Index	Group		Week	
		2	4	6
	Control	3.26±0.25	3.19±0.15	3.24±0.30
Ca^2+^ (mmol/l)	PG	3.20±0.31	3.23±0.16	3.32±0.18^(1)^
	VG	3.33±0.22	3.49±0.11^(1)^	3.27±0.19^(2)^
	Control	2.41±0.08	2.44±0.02	2.42±0.06
(HPO4)^2−^ (mmol/l)	PG	2.93±1.12	2.40±0.21	2.64±0.25
	VG	2.44±0.62	2.35±0.60	2.47±0.26

(1) Compared with the control group, *P*<0.05; (2) Compared with PG, *P*<0.05; PG, forced posture group; VG, vibration group.

### Change in serum BALP, BGP, and TRAP

Compared with the control group (8.34±0.54 u/ml), the PG group had significantly higher TRAP levels at the second week (8.34±0.54 *vs*. 85.16±1.45 u/ml, *P* = 0.032), the fourth week (8.22±0.88 *vs*. 142.00±18.54 u/ml, *P* = 0.027), and the sixth week (7.21±0.86 *vs*. 84.19±0.17 u/ml, *P* = 0.038). Significantly high levels of TRAP in VG group (180.70±101.21 u/ml) were detected in VG group, as compared to control group (8.34±0.54 u/ml, *P* = 0.034) or PG group (85.16±1.45 u/ml, *P* = 0.029). Lower BGP (12.44±2.77 ng/ml) was found in VG group (12.44±2.77 ng/ml, *P* = 0.021) and PG (9.87±2.29 ng/ml, *P* = 0.036) group at the second week, compared to control group (21.11±2.23 ng/ml). However, the levels of BGP in VG group (13.06±2.56 ng/ml) at the fourth week were markedly lower than that in PG group (31.46±29.98 ng/ml, *P* = 0.014). In addition, compared with control (10.64±0.96 ng/ml), BALP (20.41±6.15 ng/ml, *P* = 0.033) was higher in the VG group at the fourth week (Table 2).

**Table 2:** Change in serum BALP, BGP, and TRAP in the groups (mean *±* SD, *n* = 42).

Index	Group		Week	
		2	4	6
	Control	11.82±1.42	10.64±0.96	11.36±0.95
BALP (ng/ml)	PG	9.95±3.58	8.36±0.93	14.02±6.12
	VG	5.09±0.30	20.41±6.15^(1)^	12.00±0.17
	Control	21.11±2.23	18.18±1.82	19.11±2.54
BGP (ng/ml)	PG	9.87±2.29^(1)^	31.46±29.98	17.45±4.05
	VG	12.44±2.77^(1)^	13.06±2.56^(2)^	16.75±1.04
	Control	8.34±0.54	8.22±0.88	7.21±0.86
TRAP (μg/ml)	PG	85.16±1.45^(1)^	142.00±18.54^(1)^	84.19±0.17^(1)^
	VG	180.70±101.21^(1) (2)^	120.24±43.19^(1)^	73.62±1.19^(1)^

(1) Compared with the control group, *P*<0.05; (2) Compared with PG, *P*<0.05; PG, forced posture group; VG, vibration group; TRAP, tartrate-resistant acid phosphatase; BALP, bone-specific alkaline phosphatase; BGP, bone gla protein.

### The morphology of L5

The morphology of L5 was assessed by HE staining (Fig. 2). In the control group, there was no significant change in the number of osteoblasts and osteoclasts; osteoblasts arranged in a monolayer arrangement around the bone trabecula, and osteoclasts were rare (Fig. 2A). In the VG group, the number of osteoblasts was reduced, the number of osteoclasts was increased, and bone trabecular and bone structural were damaged at 2 weeks (Fig. 2B). In this group, the most serious bone injury emerged at 4 weeks, with a further increase in osteoclasts, a further significant reduction in osteoblasts, an increase in empty pit, a reduction in the bone trabecula, and structural disorder (Fig. 2C). In addition, osteoblasts were markedly reduced, osteoclasts were visible, and the marrow cavity was filled with fat cells (Fig. 2D). At 6 weeks, the number of osteoblasts was slightly increased, and osteoclasts were rare compared with at 2 and 4 weeks (Fig. 2E). In the PG group, bone injury was evident until the sixth week (Fig. 2F).

In order to count the cell number specifically, cells in a field of 500 μm×500 μm were counted. Comparison of the number of osteoblasts, osteoclasts, and the mean rate of empty lacunae in the different groups at different time points are shown in Table 3 and Table 4. Compared with the control group, the number of osteoblasts was significantly reduced in the PG group at the fourth week (89.00±3.00 *vs*. 107.00±7.81, *P* = 0.018) and sixth week (77.00±6.00 *vs*. 110.00±8.01, *P* = 0.031). The number of osteoblasts was significantly reduced in VG group compared with control groups at the second week (62.47±4.04 *vs*. 108.00±5.00, *P* = 0.029), the fourth week (71.33±4.04 *vs*. 107.00±7.81, *P* = 0.018) and the sixth week (95.00±7.03 *vs*. 110.00±8.01, *P* = 0.021). The number of osteoclasts in PG group (24.00±3.00 *vs*. 16.00±3.02, P = 0.017) was significantly increased at the sixth week. And the number of osteoclasts in VG group significantly increased at the second (26.57±1.53 *vs*. 17.00±1.00, *P* = 0.031) and fourth week (25.00±1.73 *vs*. 15.00±2.00, *P* = 0.037). Furthermore, the number of osteoclasts in VG group (26.57±1.53) was markedly increased compared with PG group (16.00±2.00, *P* = 0.017) (Table 3). The mean rate of empty lacunae was significantly increased in VG group compared to PG group or control group (all *P*<0.05). The mean rate of empty lacunae in the VG group reached 41.1±8.3% in the fourth week and drop to 32.8±7.3% at the sixth week (*P* = 0.035). It was decreased at the sixth week, but was still higher than that of the control group (*P* = 0.029). The average rate of empty lacunae in the PG group was higher than in the control group, but it was lower than in the VG group (all *P*<0.05) (Table 4).

**Table 3:** Number of osteoblasts and osteoclasts in the fifth lumbar spine (mean *±* SD, *n* = 6).

Index	Group		Week	
		2	4	6
	Control	108.00±5.00	107.00±7.81	110.00±8.01
Osteoblast	PG	96.00±6.00	89.00±3.00^(1)^	77.00±6.00^(1)^
	VG	62.47±4.04^(1) (2)^	71.33±4.04^(1)^	95.00±7.03^(1) (2)^
	Control	17.00±1.00	15.00±2.00	16.00±3.02
Osteoclast	PG	16.00±2.00	21.33±3.06	24.00±3.00^(1)^
	VG	26.57±1.53^(1) (2)^	25.00±1.73^(1)^	18.00±5.00

(1) Compared with the control group, *P*<0.05; (2) Compared with PG, *P*<0.05; PG, forced posture group; VG, vibration group.

**Table 4:** Comparison of empty bone lacuna rate (%, mean *±* SD, *n* = 6).

Group		Week	
	2	4	6
Control	13.8±1.8	14.3±1.9	13.2±1.2
PG	13.9±2.7	16.3±2.3	25.2±1.7^(1) (3)^
VG	35.6±2.4^(1) (2)^	41.1±8.3^(1) (2)^	32.8±7.3^(1) (2) (3)^

(1) Compared with the control group, *P*<0.05; (2) Compared with PG, *P*<0.05; (3) Compared with itself in the fourth week, *P*<0.05; PG, forced posture group; VG, vibration group.

### Biomechanical testing

In order to study the effect of different vibration frequencies on mechanical properties of the lumbar vertebra, 5 Hz and 6 Hz were applied in the test. Compared with the control group (819.62±6.69×10^3^ N/m), structural rigidity was significantly reduced at the sixth week in the VG group (4 Hz, 383.17±63.68×10^3^ N/m, *P* = 0.034) and the PG group (375.33±50.44×10^3^ N/m, *P* = 0.026). The max loading was significantly decreased in the VG (4 Hz) group at the second (118.71±4.50 *vs*. 183.50±0.71 N, P = 0.016), fourth (83.50±3.18 vs. 207.00±1.41N, P = 0.027) and six week (115.13±37.03 vs. 206.36±5.66 N, P = 0.023) compared with control group. The max loading of VG (5 Hz) group was significantly decreased at the fourth week (148.25±15.13 vs. 207.00±1.41 N, *P* = 0.034). Meanwhile, The max loading of VG (6 Hz) group markedly was reduced at the fourth week (147.71±13.94 vs. 207.00±1.41 N, *P* = 0.029) (Table 5).

**Table 5:** Change in biomechanics in lumbar bone in the groups (mean *±* SD, *n* = 6).

Index	Group		Week	
		2	4	6
	Control	723.52±20.48	897.18±37.77	819.62±6.69
	PG	616.06±46.91	613.10±19.25	375.33±50.44^(1)^
SS (×10^3^ N/m)	4 Hz	532.1±106.12	525.31±89.09	383.17±63.68^(1)^
	5 Hz	556.29±127.23	567.14±120.98	614.06±137.42
	6 Hz	613.26±28.77	868.05±218.83	663.03±139.27
	Control	183.50±0.71	207.00±1.41	206.36±5.66
	PG	180.94±4.15	179.8±16.18^(1)^	148.8±28.27^(1)^
ML (N)	4 Hz	118.71±4.50^(1) (2)^	83.50±3.18^(1) (2)^	115.13±37.03^(1)(3)^
	5 Hz	125.94±9.99	148.25±15.13^(1)^	178.03±23.20
	6 Hz	132.50±41.29	147.71±13.94^(1)^	196.75±19.45

(1) Compared with the control group, *P*<0.05; (2) Compared with PG, *P*<0.05; (3) Compared with itself in the fourth week, *P*<0.05; PG, forced posture group; VG, vibration group; SS, structural stiffness; ML, Max loading.

## Discussion

In this study, we demonstrated that forced posture and vibration can cause pathological changes to the lumbar vertebra, and vibration combined with a seated posture could cause further damage to bone metabolism. Vibrations led to the cell numbers alterations of osteoblasts and osteoclasts in lumbar vertebra which provided evidence that vibrations was associated with functions of bone cells. Furthermore, we also found high frequency of vibration caused more great damages.

BALP, BGP and TRAP are secreted by osteoblasts and osteoclasts which are not only the specific indicator of bone metabolism but also the specific experimental diagnostic indicators for bone remodeling evaluation [23]. BALP is a glycoprotein ecto-enzyme linked to the osteoblast membrane which is released into circulation during the bone-forming phase of the remodeling process [19]. BGP is a specific marker of osteoblastic bone formation, the serum level of which shows positive correlation with the synthesis of osteoblasts [24], [25]. TRAP is secreted into the circulation by osteoclasts which has been proposed as a marker enzyme of bone-resorbing osteoclasts [23]. TRAP functions as an osteopontin phosphatase in bone resorption, and may also act as a modulator of resorption activity due to its secretion kinetics [26], [27]. In this study, compared with the control group, TRAP was significantly increased at the second week, whereas BGP was decreased at the second week in the VG group, indicating that early vibration resulted in increased bone resorption. Although BALP was increased at the fourth week, the bone remodeling did not exceed bone resorption indicating the delayed bone remodeling. TRAP was reduced at the sixth week, but still high compared to control group indicating that the bone remodeling was still uncompleted.

Further studies using HE staining showed that the number of osteoblasts was decreased and osteoclasts was increased, and bone trabecular and bone structural were damaged at the second week. The most serious bone injury emerged at the fourth week, which was slightly alleviated at the sixth week, indicating that vibration was closely related functions of bone cells. It has been reported that fatigue microdamage coincides with osteoclastic bone resorption [28]. Our findings show that the mechanical properties of the lumbar spine are affected by vibration and/or forced posture. Forced posture and the vibration might induce microdamage which initiates bone resorption. In this study, the effect of vibration in a seated posture (simulating HEV operators), which can result in LBP on bone metabolism, biochemical bone metabolism indices, and morphometric and mechanical properties of the lumbar vertebra were studied. From the results, we can clearly see that vibration in a forced posture (simulating HEV operators) can cause disorder of bone metabolism and bone damage, which is one of the causes of LBP. The vibrational frequency (4 Hz) caused the biggest damage to the lumbar vertebra. Therefore, in daily life and at work, we should employ vibration reduction (especially avoid 4 Hz) and avoid sedentariness.

However, it's intriguing that evidence has demonstrated that short-term whole body vibration training leads to no changes in none of the bone mineral content and bone mineral density parameters, but decreases the tibia, total, trabecular and cortical volumetric bone mineral density, which improves the bone mass and structure in elderly people [29]. In rat model, whole body vibration improve the bone loss induced by ovariectomization [30]. Whole body vibration training alone or in combination with exercise help to increase or at least prevent decline in bone mass with ageing, especially in postmenopausal women [31]. These results seemed contradictory to our findings, however the underlying mechanism of vibration in bone metabolism might be involved in multiple factors and intrigued. For example, the animal models in our study were received vibration in forced posture that might cause different effects. It might be because of occupational vibration directly affecting the trunk. Also, occupational vibration causes LBP that might be partly because of the forced sitting posture which was uncomfortable with long time.

## Conclusion

This study demonstrates that bone injury caused by forced posture and vibration is closely related to bone metabolism. The indicators of bone remodeling and resorption can be potentially used as early markers for clinical diagnosis of the bone-derived LBP. Furthermore, the forced posture and vibration is more like the driver's environment, which is of important research significance. However, the underlying mechanism deserves further research.
